# Views from many worlds: unsettling categories in interdisciplinary research on endemic zoonotic diseases

**DOI:** 10.1098/rstb.2016.0170

**Published:** 2017-06-05

**Authors:** Hayley MacGregor, Linda Waldman

**Affiliations:** STEPS Centre, Institute for Development Studies, University of Sussex, Brighton BN1 9RE, UK

**Keywords:** anthropology, zoonotic disease, human–animal relations, One Health

## Abstract

Interdisciplinary research on zoonotic disease has tended to focus on ‘risk’ of disease transmission as a conceptual common denominator. With reference to endemic zoonoses at the livestock–human interface, we argue for considering a broader sweep of disciplinary insights from anthropology and other social sciences in interdisciplinary dialogue, in particular cross-cultural perspectives on human–animal engagement. We consider diverse worldviews where human–animal encounters are perceived of in terms of the kinds of social relations they generate, and the notion of culture is extended to the ‘natural’ world. This has implications for how animals are valued, treated and prioritized. Thinking differently with and about animals and about species' boundaries could enable ways of addressing zoonotic diseases which have closer integration with people's own cultural norms. If we can bring this kind of knowledge into One Health debates, we find ourselves with a multiplicity of worldviews, where bounded categories such as human:animal and nature:culture cannot be assumed. This might in turn influence our scientific ways of seeing our own disciplinary cultures, and generate novel ways of understanding zoonoses and constructing solutions.

This article is part of the themed issue ‘One Health for a changing world: zoonoses, ecosystems and human well-being’.

## 1. Introduction

The ‘One World One Health’ agenda stresses the interconnections between humans, animals and the environment and calls for integration and collaboration between veterinary and human medicine [1]. It is also interpreted at a global institutional level as promoting greater collaboration between international agencies with oversight of human and animal health, as well as agriculture and food. The rise of this agenda has been linked to the growing recognition of zoonotic diseases as a global challenge [2]. An estimated 60% of human infectious diseases and approximately 75% of emerging infectious diseases are zoonotic [3]. With respect to research, there have been calls for interdisciplinary consortia to tackle these issues and recognition of the need for integrated frameworks for the study of zoonoses [4–7]. Funding calls for zoonotic disease research now frequently presuppose interdisciplinary, and indeed transdisciplinary, collaboration.

In this paper we reflect on the contribution of anthropological and broader social science perspectives in interdisciplinary research, in relation to neglected, endemic zoonoses. These tend to involve the livestock–human interface, although wildlife systems can be implicated as well. In low- and middle-income countries, endemic zoonoses severely affect poor livestock producers whose livelihoods are dependent on animal husbandry [8]. We draw on our research as anthropologists within interdisciplinary consortia focused on the transmission of zoonoses in livestock systems and also on exploring the risks of disease emergence.

We begin by considering existing work on zoonoses that has uncovered practices that facilitate the spread of organisms from animals to humans and might increase the risk of disease. There are pertinent reasons for using disease risk as a departure point in interdisciplinary studies of zoonoses, and social science research has contributed to the identification of points of transmission of pathogenic zoonotic organisms. We wish to suggest, however, that a further contribution could be made by social science involvement in interdisciplinary research through extending beyond an emphasis on risk. An expanded contribution could draw in perspectives from the anthropology of human–animal relations, and an increasing attention to the study of the non-human, for instance within anthropology, geography and science and technology studies. This is a large body of work and we have not attempted an exhaustive review. Rather, we have sought to draw upon examples of this work that are pertinent to endemic zoonotic disease. We propose that consideration of this body of scholarship can provide a more nuanced and expanded account of the human–animal interface, moving beyond a conceptualization of ‘interactions’ between people and livestock to consider also the social relations and social practices that mediate porous boundaries between humans and animals in fluid and contingent ways. Furthermore, the body of scholarship on human–animal relations extends a well-established tradition in anthropology that, by drawing on comparative ethnographic perspectives across cultures, questions the universality of conceptual divisions presumed in Western thought, most fundamentally (and most pertinent to this discussion) that of ‘nature’ versus ‘culture’ [9]. Drawing these perspectives into interdisciplinary work could productively unsettle the categories implied by ‘One Health’ frameworks and the boundaries readily drawn between humans, animals and the environment. Such categories and the way in which boundaries are conceptualized also determine, and are in turn determined by, the ways in which disciplinary cultures construct knowledge, for example in scholarship on zoonotic diseases. The categories tend to be mirrored in the constitution of interdisciplinary consortia for work on zoonoses, which commonly include veterinarians, human health experts and disciplines with an ecological remit.

By drawing attention in this paper to a cross-cultural literature on the human–animal interface and reflecting on its relevance for understanding and tackling endemic zoonoses, we hope to prompt more engagement with the implications of different worldviews and spiritual ecologies for understanding human–animal relations and the boundaries between species. We thus argue for a deeper analysis of culture and social practice to be included in interdisciplinary dialogue and in the framing of research. Such analyses would illustrate the diversity of views regarding the nature of the human–animal interface and of relations between animals and humans and explore how people in different contexts understand animals' social value, health and welfare. These views could correspondingly affect how people might respond to animal illness and measures to control zoonoses. Our reference to ‘views from many worlds’ does not arise from an essentialist assumption of multiple, bounded cultural worlds. Rather, it is a call for scholarship arising from interdisciplinary collaborations on endemic zoonoses to include field enquiry that explores the diversity of worldviews regarding the nature of animal–human relations and the fluidity of bodies and boundaries. This would complement existing scholarship that seeks to embed understandings of disease risk within a wider political economy, and contribute strategies for the design of more effective interventions for zoonotic diseases that are acceptable to local people. Moreover, an anthropological critique of categories assumed in Western thought, and recognition of the value of understanding human–animal relations as situated in a broader social field, could open up fresh exchanges across the disciplines involved in One Health agendas, and productively destabilize assumptions. It could transform how the interests of people in research sites are understood and enrich researchers' engagements with them, thus advancing a shared agenda for the prevention and control of zoonotic diseases, both in outbreak situations and when addressing neglected endemic zoonoses.

## 2. Interdisciplinary research on zoonoses: understanding ‘risk’

In interdisciplinary research on zoonotic disease, it is a common to conceptualize the issues in terms of ‘risk’, such as the risks associated with disease emergence, or factors increasing risk of transmission or spread. Frameworks proposed for work on livestock and wildlife systems and zoonotic disease have thus emphasized the ‘risk environment’ and ‘risk scenarios’ for transmission of organisms and the importance of understanding drivers of risk at different scales (see for example [7]). Concomitantly, there are usually expectations for the social science dimensions of research to contribute information regarding the cultural beliefs and practices that influence human–animal ‘interactions’ and livestock keeping, with respect to reducing risk and contributing to the control of (re)emerging and endemic zoonoses. For example, work in Laos has contributed valuable data by uncovering the practice of eating raw pork and elucidating its relationship to masculinity and also to the risk of *Taenia solium* [10]. Woldehanna & Zimicki [11] have developed a framework to analyse the social and environmental factors that contribute to disease exposure in a similar context. There has also been social science engagement with the risk of emerging zoonotic disease, such as the critical insights gained regarding Nipah virus spread in Bangladesh [12].

Of course there are very real public health, economic and social rationales for emphasizing risk reduction, so it is not surprising that risk is often a central practical and conceptual focus of interdisciplinary research. However, an exclusive focus on risk in studying the social dimensions of zoonoses increases the likelihood that, in interdisciplinary consortia, people's cultural logics or social practices come to be cast in negative terms, and as beliefs or behaviours that exacerbate risk and require changing. Such an approach can restrict social science dimensions of zoonotic disease research to understanding the context from which risk emerges, or to facilitating behavioural change. In outbreak situations, the worldviews and social responses of local people can readily be cast as ‘risky behaviour’, ignorance or superstition, and as generating ‘unnecessary’ resistance to control efforts [13]. In interdisciplinary public health research more broadly, social scientists have commented on the need to counteract a view of ‘culture’ as fixed, immutable beliefs and practices that perpetuate disease risk [14] and to ensure that the incorporation of social science findings are not reduced to informing strategies for achieving individual behavioural change, as if this endeavour involves a simple linear process of social engineering [15,16].

Scholarship has contributed to broadening understandings of disease risk beyond the individual by including an analysis of the wider political economy of zoonotic disease in different contexts (see for example [17]). Such analysis draws on recognition of the ways in which the organization of society and conditions of inequality can influence the risk of infectious disease, particularly for marginalized groups, as a form of structural violence [18]. Attention has also been given to the way in which an intersection of social difference and marginality can increase the risk of disease, for example the intersection of poverty and gender affecting risk of *Taenia solium* in an endemic area in Eastern Zambia [19]. Such work informs a nuanced understanding of how people experience and indeed conceptualize risk within a wider web of structural factors and intersecting inequalities, and how cultural beliefs and broader understandings of health and well-being might affect the construction of knowledge regarding zoonoses (including the degree of risk that these diseases are seen to pose in local contexts). This allows appreciation of the socio-economic and political contexts in which behavioural change interventions might be located and enables these to be conceptualized in relation to structural factors that can limit individual agency. Indeed, in contexts of fragile livelihoods, disease risk might not be people's sole concern or they might not have the resources to take up protection measures. People might even be willing to accept a disease risk as they weigh-up trade-offs, such as between their economic well-being and potential harms to their physical health. They might also resist zoonosis control efforts that directly threaten their broader well-being. There are, for example, compelling accounts of the effect on the livelihoods of small farmers from mass culling policies as a measure against avian influenza (see for example [20,21]).

A rethinking of the risk paradigm as it influences international policy and response has been pursued in analyses of different framings of zoonoses [22]. These illustrate how particular understandings of risk focused on fear of outbreak can ‘close down’ responses and exclude more open, deliberative pathways of response based on an appreciation of uncertainty [23,24]. These analyses enable a critical but constructive contribution by pointing out the limits of disease modelling and surveillance and of narrow, singular policy approaches. Thus scholarship unpacking diverse understandings of risk, from perspectives on the ground all the way up to the level of policymakers, constitutes an important contribution to interdisciplinary work on zoonoses, given the centrality of risk in this field.

## 3. Human–animal relations and expanded social worlds

In this section we consider another body of social science scholarship, one which examines more closely the categorization of society (humans) and of nature (animals) and unsettles the assumption of immutable boundaries. We argue that this work might enable a fresh and complementary perspective to the study of zoonotic disease by allowing an entry point to the study of human–animal–environment relations that does not only take risk as the point of theoretical departure for studying the social dimensions of zoonoses. Moving beyond a perception of humans and animals as two distinct categories enables a shift also from a focus on ‘interactions’ between them to consider an expanded purview of social worlds.

Within anthropology it has long been known that societies have diverse understandings of animals. Animals are ‘good to think with’, said Levi Strauss (in Leach's [25] translation) or, as Donna Haraway evocatively suggested, ‘we polish an animal mirror to look for ourselves’ [26, p. 21]. This is because animals' different shapes, characteristics and behaviours can be applied to humans—who have less obvious physical differences—and used to conceptualize social relationships and boundaries. As such, particular animals come to assume symbolic and political meaning [26,27]. In recent years scholarship in anthropology has demonstrated a renewed interest in the subject of human–animal relations [28,29]. In reconsidering this field, anthropologists have moved away from an earlier tendency to see animals as merely of economic use to humans, of ritual significance, or as a means to ‘think with’ and reflect on the organization of human society [27,30]. Anthropology as a field has an interest in revealing other kinds of realities, such as creating awareness of different understandings of the nature and categories of being, or different ontologies. Thus with respect to human–animal relations, anthropologists have paid increasing attention to alternative ontological realities or ways of being that assume a fluidity in species' boundaries [31,32]. Conceptualizations of animals and humans as interdependent, views which do not take for granted the notion of bounded species categories or indeed that of bounded bodies, contrast with ‘the reality’ as perceived within biomedical and veterinary worlds. A focus on the interlinking of human and animal realms (and indeed the realms of microorganisms, insects and so forth) is also quite distinct from an approach that sees contact with animals as a set of interactions that primarily create risk to humans. If human–animal relations are recognized as part of the social world, then these need to be managed or negotiated, with a different level of connection, and of attention. The notion—and separateness—of the ‘natural’ no longer holds as an unquestionable given, as modalities of co-existence across species' boundaries are explored [33]. Different species can be seen as ‘simultaneously actors and participants in sharing and shaping mutual ecologies’ [28, p. 600]. These social ecologies are also subject to changes, from factors such as land-use or climate change [28].

Two examples of such anthropological scholarship will serve to demonstrate this interest in human–animal relations and the alternative perspectives it reveals: the first body of work relates to the polar north, and the second focuses on the cosmologies of the Amazonian region in South America. Both pursue a longstanding anthropological interest in hunters and the ontologies that perceive of hunting as a form of exchange between human and non-human persons [34]. These views unsettle notions of ‘domestic’ and ‘wild’ and serve as good examples to explore the complexity of different understandings of the cohabitation of humans and animals in shifting relations of closeness, from livestock to prey. We explore these examples at some length to illustrate how human/non-human engagements are culturally shaped and understood in social terms, and to point to the implications that such beliefs might have for categories that are taken for granted in the medical worlds that shape One Health discourse and research.

With respect to the polar regions, Ingold [35,36] has documented a dynamic landscape of reindeer–human relations over a prolonged period in the Siberian north. He examines relationships between man and deer, and an understanding rooted in accepting deer as social and decision-making animals. He proposes that both men and reindeer view this as a ‘transactional’ relationship in which both benefit. Men use the animals in order to sustain their livelihoods and ecosystems. At the same time, reindeer are able to recognize that men can protect them from different threats in different seasons (predatory animals, mosquitoes, hunger) and thus voluntarily socialize with them. These transactions, Ingold argues, are based on each groups' recognition of the existence of the other, which leads them to interact based on decision-making that considers the respective value of the other and maximizes this. The domesticated deer is part-socialized into a human environment. The herder possesses clear ownership rights over his deer, but his control is based on a form of social contract between man and deer.

Ingold examines how these dynamics have been interrupted by changes in the ecosystem (natural and human induced), which leads to the introduction of new technologies which break the transactional nature of the relationships between men and deer. As opposed to the scenario of socialization, the reindeer cease to recognize obligations towards their masters. In this new sociocultural and economic context, which Ingold calls predatory pastoralism, control over the reindeers can only be regained through physical force and technological superiority and is characterized by roles of pursuer and pursued. He argues that this has negative effects on human economic and social well-being.

Willerslev [37] offers an analysis of Siberian Yukaghir hunters that further explores an empathetic dimension of social engagement with animals that does not presume that animals are ‘wholly natural kinds of being’ (p. 629). They are considered to have attributes of personhood, such as moral responsibility and intentional (not only instinctual) behaviours. Central is the assumption that humans are not the only ‘persons’. Personhood can be situated in rivers, trees and mammals: ‘[i]n their world, persons can take on a variety of forms, of which human beings are only one’ (p. 629). Moreover, humans and animals can change into each other, so that a hunter can, for a period, assume the way of seeing of his animal prey, while not literally becoming the animal.

In Amazonian anthropology, Vivieros de Castro has advanced the theory of ‘perspectivism’ [38] through an analysis of the understandings of the Amazonian indigenous population (Amerindians) regarding the bodies and souls of human and non-human ‘persons’ (animals, spirits), and the relationship to the surrounding environment. He demonstrates how humans and non-humans perceive each other, and interact as beings who possess souls. These souls, rather than physical bodies, are the basis for interaction. This aspect of Amerindian thought demonstrates a ‘perspectival quality’, a conception in which the world is inhabited by different sorts of subjects or ‘persons’, human and non-human, who perceive reality from distinct points of view. Significant relational positions would be those of predator and prey.

Vivieros de Castro considers points of merging between animals and humans, such as in rituals. The understanding that a soul might move between bodies allows a shaman in rituals to assume the perspective of a jaguar. Inhabiting a particular body then enables and differentiates a perspective and the identity that it brings. By focusing on the soul, Amerindians establish boundaries and ways of engaging with non-human subjects/persons. Importantly, the ethnographic point of view described by Vivieros de Castro [38] defines ‘an ontology which postulates the social character of relations between humans and non-humans: the space between nature and society is itself social’ (p. 473). He contrasts this perspective with a Western ontology, where ‘the nature/society interface is natural: humans are organisms like the rest, body-objects in ‘ecological’ interaction with other bodies and forces, all of them ruled by the necessary laws of biology and physics’ (p. 473). In Western ontology then, social relations are confined to the realm of human society.

These ethnographic examples show how scholarship in the anthropology of human–animal relations draws our attention to cosmologies that do not conceptualize binary categories (such as human:animal and culture:nature) as fixed and universal, as is the dominant understanding in Western philosophical thought. Indeed, in the particular ontological realities presented above, human–animal encounters are perceived of in terms of the kinds of social relations they generate, and the notion of culture is extended to the ‘natural’ world. This has implications for how animals are valued, treated and prioritized [1], which in turn has implications for the problems of zoonotic disease control. Such accounts of the assumed sociality of human–animal relations in different contexts challenge the centrality of ‘risky behaviours’ in the dominant framings of research on endemic zoonoses. Furthermore, as Vivieros de Castro points out, a view of humans and animals as biological organisms has particular philosophical roots. It is this worldview that dominates zoonotic disease research. Thus if we are to take seriously the possibility that people in the places where we do research might not see things in the same way, then we have to be willing to have our categories unsettled and to grapple with the practical implications of this for engagement in field sites, for knowledge sharing, and for the design of interventions.

## 4. A multi-species perspective and zoonotic diseases

There is a growing body of scholarship that has recently begun to apply these insights to the consideration of the human–animal–microbe worlds, including scholarship on the microbiome, on vector-borne disease and on zoonotic diseases with epidemic potential. Craddock and Hinchliffe [2] point to inadequate engagement by the social sciences in studies of One Health and disease so that the potentially productive potential of these insights remains underexplored. Wolf [39] notes that animal–human engagements have been limited to ‘natural-cultural’ domains and frequently conceived of as biological phenomena in studies of disease. She argues for a re-orientation to situating analyses of zoonoses within the sociocultural domain, focusing in particular on the way in which consideration of the microbiome unsettles a notion of a bounded human body. Hinchliffe argues too that social relations between humans and animals, and correspondingly the diverse ways in which people know and explain their experiences, have been ‘lost from view in one health’ [40, p. 30]. It is this knowing, and these relationships, which sustain intense and repeated animal–human interactions, and in so doing, create the conditions for ‘viral chatter’ and contamination ([29],[40, p. 30]).

With respect to studies of viral haemorrhagic fevers, Brown and Kelly [41] argue for an extended conceptualization of the social by considering the anthropology of human–animal relations. Through an analysis of material practices enacted in different social spaces, they question assumptions of rigidity in categories of ‘wild’ and ‘domestic’ with respect to understanding how people live with and relate to rats and how they balance this living with rats against the risk of Lassa fever. Cassidy [31] has pointed out that farmers, caught up in the British bovine spongiform encephalopathy disease control efforts, had a very different relationship with their animals that unsettled the divisions of nature and culture assumed in control efforts. Singer [42] has extended his notion of ‘syndemics’—when interactions exacerbate the negative effects of one or more diseases in a society—to include zoonotic outbreaks: he proposes ‘ecosyndemics’ as an expanded concept that can characterize the biosocial engagements that coalesce in disease transmission. Interspecies relationships—close human relationships and regular intimate interactions with animals and zoonotic pathogens—have over generations created and sustained, and will in future further create and sustain, the potential for ‘spillover’ of organisms across species' barriers. These mutually causal relationships mean that humans and domestic animals evolve and change concurrently [42]. This realization thus becomes as central to understandings of zoonotic disease transmission as changing biological phenomena. Indeed the domains are intertwined, with important implications for control of zoonoses.

## 5. Livestock–human relations: the example of cattle in east and southern Africa

In interdisciplinary research on endemic zoonoses, the insights from scholarship on human–animal relations are still largely absent in the framing of research. In this section, we illustrate how an expanded understanding of the interconnected worlds of humans and animals is evident in existing ethnographies. We then focus on the implications for work on endemic zoonoses and consider what a broader conceptualization of the social—in terms of risk and also of human–animal relations—can bring to research on zoonoses. As the example shows, appreciation of the social value of cattle has particular implications for understanding livestock-linked zoonoses in the African context.

Among the pastoralists in southern and eastern Africa, cattle have been of primary symbolic importance. As first noticed by Evans-Pritchard working among the Nuer in the 1960s, cattle are not just draught animals of a certain economic worth, nor are they simply a supply of regular meat. People's lives have been closely entwined with their cattle at economic, political, social, relational and everyday interactional levels. In southern Africa, cattle were—and often continue to be—markers of gender, status and power and, as such, could not be easily commoditized or given a monetary value. Indeed, among the Tshidi Barolong, keeping cattle could be seen as a form of resistance against a commoditised economy [43]. Cattle have been—and continue to be—profoundly associated with social relationships. Because cattle were (and still are) used for bridewealth, they linked men and women into communities in particular ways, represented men's lineages, legitimated children and determined access to labour. Moreover, cattle provided economic support for families, offered a means of sharing resources across households and of building a presence in times of physical absence. Through exchanging animals and distributing animals to households in different places, recipients were (and are) able to benefit from the cattle (milk, calves, labour) and from owners' investments into the cattle (payments for labour). Cattle therefore provided ‘the means to engage in and maintain social networks and circuits of exchange’ that stretched between rural households and across rural and urban space ([44, p. 136],[45]).

In east Africa, cattle have been and still remain significant as social, political and symbolic entities. The skill of Maasai herders to intimately know large numbers of cattle—in terms of recognizing and classifying individual animals, and recalling each animal's genealogy and ownership history—has been celebrated [46]. Cattle formed a central component in rituals and were/are closely linked to people, symbolizing people and societal relationships and being sacrificed to promote human life and resolve social conflict [47]. For example, a Nuer man's adult name was drawn from one of the oxen he received at his initiation and age-sets were named after the oxen sacrificed at their Eunoto ceremony (representing the conclusion of warriorhood and promotion to the next age-set). The ox, and the ritual associated with its sacrifice, epitomized and linked together all the individuals in the age-set, as well as symbolizing the age-set as a social collectivity and its relationships to broader society. More generally, societal divisions echoed distinctions in cattle herds (cattle-with-dark markings, black cattle) and references to individuals as cattle (my ox, my calf, my heifer) were both a form of endearment and a recognition of social roles [46]. As in southern Africa, material conditions, cattle and collective meaning were—and continue to be—deeply entwined [48] and cattle provided a means for marginalized people to ‘integrate into their social, cultural and linguistic universes, the often catastrophic changes that they have experienced over the past six decades’ [44, p. 132]. Cattle were, and remain, more than meat and animal labour, they were and are stores of cultural value. In these regions of Africa they symbolize and sustain society.

## 6. Livestock–human relations and understanding neglected endemic zoonoses

Wolf [39] points to the significance of the social determinants of human–animal engagements in approaching One Health problems. In the example of cattle–human relations, and considering the consequences of this reality for understanding endemic zoonotic disease, this insight can be extended. Galaty, working among the Maasai, suggests that social relationships between humans and animals go beyond seeing animals as symbolic of human society: ‘[f]or pastoralists, livestock lie within the domain of sociality and are individuals, with names, personalities, genealogies, and social ties’ [46, p. 34]. Not only do people know, think about and interrelate with cattle, cattle know and think about people, shaping their interactions accordingly. Galaty thus describes a situation where, not only are animals seen as symbolic or representative of human differences, but there is a ‘meshing of human and animal identities’ in that domestic animals are an integral part of pastoralist society [46, p. 31]. Similarly, Abbink [47, p. 342], exploring Suri society in southwest Ethiopia, argues that cattle are the ‘essential precondition’ for human society. Vital relationships exist, not just between human members of pastoralist society, but also between humans and animals. There is, in other words, a sociability of animals [46,47]. Domestic animals engage and respond to humans just as much as humans engage and respond to animals. Among the Maasai, domestic livestock are conceptually included in the realm of humans and homesteads, and contrasted with the forest or bush and wild animals [46]. Moreover, Maasai cattle—like Maasai—are individuals, with particular personalities, names, lineages and social connections and cattle are socialized: calves are kept inside Maasai houses, cattle are constantly monitored, touched and communicated with (in animal–human speech, calls, whistles, songs).

It is thus evident that a deeper engagement with understandings of human–animal relations from different cross-cultural contexts requires us to rethink how people in different parts of the world consider their animals in general, but also more specifically in terms of the animals' health, welfare and diseases that can be harboured. Such an expansion of the domain of the social could add considerable nuance to interdisciplinary research. As Brown *et al.* [49] argue, social relationships and, we would add, intimacies are felt to exist between animals and humans. These significantly affect how animals are valued and managed and also the potential for zoonotic disease spread. Animals are not necessarily conceptualized as different from humans. As such, health information messages to treat animals in particular ways can be open to misinterpretation, and may be dismissed outright as impossible or inhumane.

Those leading interventions for control of zoonoses should also not make quick assumptions about who has decision-making power with respect to livestock keeping. Social norms influence how people interact with livestock and other animals, shaping both who is exposed to pathogens and who interacts with what kinds of animals [11]. While one person may be in charge of an animal on a day-to-day basis, decisions about the welfare of the animal may be taken by someone else, who is not physically present. Or, despite seemingly clear cultural norms on who is nominally responsible to make such decisions about animals, negotiation and contestation may result in far less predictable outcomes [44]. Decisions about animal treatment and management are not straightforward and seldom the domain of one person. Rather, they may be spread across geographical space (if animal owners live far from animal carers) and time and highly contested. These dynamics have to be unpicked to determine who should be targeted by zoonosis control information messages and to understand who has the power to decide whether to implement suggested campaign strategies or comply with regulatory measures.

Using animals as a means to ‘think about’ zoonoses makes it evident that social relationships between humans not only inform animal–human interactions, but also influence decisions about long-term strategies of animal management. And as human relationships and hierarchies shift, so can these strategies. In the example of pastoralists, scholarship shows that women and young people are, in a range of ways, contesting senior men's ‘ownership’ of and control over cattle in complex and unanticipated ways and shaping decisions about whether and how animals are kept, dispersed to other households, sold or slaughtered [44,50]. These ‘complex entanglements between humans, environments and pathogens’ [39, p. 5] have to be disentangled and understood from the perspective of both human–human and human–animal relationships. Ethnography can reveal the complexity of such entanglements and bring to the fore the implications for the value livestock owners' place on their animals, for approaches to animal welfare, and for the priority placed upon animal health.

This broader understanding of the social and cultural shaping of human–livestock relations and of how people value their animals is also critical for a perspective on zoonoses that places disease risk in a broader context. How people think about and decide what to do about a sick animal, for example, may not be influenced only by decisions about economic gain or about potential disease spread. Insights regarding the relations with animals have to be considered alongside an understanding of political economy and people's situations with respect to livelihood options and access to human and animal healthcare. Poor people in particular have to make difficult and pragmatic decisions about livelihoods, and livestock are integral to these. For example, the fact that cattle hold great symbolic importance and are connected intimately to social life in east and southern Africa, does not mean that cattle are not slaughtered, sold or substituted for cash. However, as failed attempts to promote cattle markets for economic development have grudgingly come to realize, it does mean that pastoralists' rationales for doing so, and the arguments made by outsiders promoting development initiatives, are often very different [44,45].

Risks, as conceptualized by zoonotic disease specialists, are not necessarily the same as risks conceptualized by livestock owners. Avoiding one risk (by treating diseased animals) may expose people to other risks (reduction in ability to sustain livelihoods; inability to pay bills for animal treatment; extended family members' anger as ‘inappropriate’ decisions are made by the animal keeper). To consider an example from another region of the world, backyard or small-scale pig production constitutes an important form of livelihood for many farmers or peri-urban dwellers in East and Southeast Asia. Pigs can be an insurance against debt and their sale a source of cash for unexpected expenses. Pork tends to have a high social value and in this part of the world is the food for important festivities such as the New Year. Yet the risks of endemic zoonoses and of disease outbreaks is thought to be increasing with rapid intensification of pig production in contexts where regulatory and biosecurity measures remain weak. Research in Vietnam, for example, has focused on the risks, including to particular social groups [51].

Dynamic global drivers of change in livestock systems shape political and economic structures that affect poor farmers and those reliant on livelihoods in animal supply chains. Those operating with narrow profit margins and limited access to credit or compensation for dead animals, are constantly faced with decisions involving difficult trade-offs, such as between attending to the health and productivity of their animals, the costs of agricultural inputs, and the economic viability of their farms. These trade-offs can enhance the scope for zoonotic disease spread. Understanding of macro-level drivers constitutes an important part of analysing such micro-level dynamics.

Neglected endemic zoonoses are transmitted in situations where people live in close proximity to animals and sanitation is compromised [52]. In such situations, such as with small-scale pig production, the health of animals is intricately connected to people's own health. Just as pigs' social value as a component of ‘home’ might be significant, so also human versus animal health and illness might not be conceptualized as necessarily separate. Research in endemic zoonoses tends to assume separation of human health-seeking trajectories from strategies to address livestock illness, and of human and animal health systems. In practice, these divisions might not be perceived of as rigid, and indigenous and lay healers might give assistance across domains. In settings where state public health systems are weak and resources limited, where the influence of allopathic medicine is less pervasive, and informal markets for health predominate, the integration of strategies and decision-making to address human and livestock health can happen by default. The health systems are not necessarily seen or experienced by people as distinct. People do not necessarily have access to separate human and veterinary health systems, but experience this interconnectedness also when they go to buy drugs from the drug seller, or address all illness, human and animal, through self-management strategies or indigenous and lay healing systems. Thus interventions to reduce the risk of disease have to go far beyond a focus on farmer behaviour when considering entry points for change. What is required is a more nuanced understanding of the value of animals and the trade-offs people make as they face a multitude of risks in real world situations. In addition to understanding these dimensions, we should be prompted to see beyond our own cultural and disciplinary assumptions and pay attention to a perspective that might suggest different categories, more fluid boundaries, and varied ways of being in the world.

## 7. Views from many worlds

What could the inclusion of a wider range of social knowledge add to the conceptualization of One Health? Scholarship has focused on the political economy of One Health [53] and on the disjunctures between policies and realities of implementation on the ground. However, there is still limited scholarship that rethinks the very categories assumed and taken for granted by One Health discourses. Wolf [39] points to the need to rethink assumptions of the ‘global’ and of a singular ‘one health’. Hinchliffe [40] questions assumptions of the singularity of ‘one worldism’. We argue for consideration of a richer range of cross-cultural perspectives that can bring nuanced views from ‘many worlds’ to scholarship on One Health and can expand the scope and depth of enquiry. This involves openness in interdisciplinary research on zoonoses to deeper engagement with scholarship that questions assumptions about rigid categories of human: animal and nature:culture and the ways in which these boundaries are drawn.

One Health ideas are based on an assumption of interconnectedness and intersectoral interaction, but this must be taken a step further to acknowledge ontologies where relevant categories such as of nature and culture are perceived in radically different ways. This involves closer attention to people's own knowledge about the nature of relationships between humans and animals, as well as to the implications of how people ‘think with’ and ‘think about’ animals in different contexts. If we can bring this kind of knowledge into One Health debates, we find ourselves with a multiplicity of worldviews where we cannot presuppose bounded categories, and where the interfaces and interactions between these can be reconceptualized in terms of the social and relational. This might in turn influence our scientific ways of seeing our own disciplinary cultures, enabling fresh conversations and an unsettling of taken-for-granted assumptions and boundaries.

Our own experience of interdisciplinary collaborations suggests that as relationships across disciplines strengthen over time, and in an environment of mutual respect and interest to learn from the other disciplines, it is possible to shift the commonly held and limiting perception of the anthropologist as broker, mediating across different beliefs and cultural practices with the aim of reducing disease risk. This opens new possibilities for expanding the remit of social science enquiry so that a broader social field is considered relevant to the research, and conceptual frameworks for such collaborations can gain new dimensions. There is already some indication that anthropological research on human–animal relations is being considered in interdisciplinary collaborations, at least in the example of viral haemorrhagic fevers [49]. The benefits of an expanded enquiry are not just in the potential for novel ways of constructing solutions. Greater attention to the understandings and interests of local people who are most affected by zoonoses in countries where research is done, can enhance appreciation of their interests and increase the likelihood that disease control is a shared endeavour and control measures are acceptable to all. It might thus give cause for us to pause and ask more seriously: whose knowledge counts, and indeed whose health counts?

## 8. Conclusion

We have argued for bringing a broader sweep of disciplinary insights from anthropology and other social sciences to bear in interdisciplinary conceptualization of neglected zoonotic diseases and of related One Health responses. With a focus on interdisciplinary research on endemic zoonoses in livestock, we argue that social science contributions have assisted in contextualizing risk, introducing understandings of the broader drivers of zoonotic disease and of the context of responses. We also suggest that this engagement could be extended by consideration of anthropological scholarship on human–animal relations and an unsettling of the categories central to One Health discourse. By considering scholarship from the burgeoning field in anthropology and beyond on human–animal and multi-species relations, we suggest that this scholarship could provide fresh avenues for deeper engagement with local cultural realities in work on endemic zoonotic disease. Such intellectual engagement could also enable a deeper examination of the way in which One Health discourses construct separate and discrete categories of human–animal–environment and to think more seriously about how the interactions between these are at present conceptualized as compartmentalized. Thinking differently with and about animals and about species boundaries may help to generate novel ways of addressing zoonotic diseases which have closer integration with people's own cultural norms and understandings of human–animal dynamics.
